# Description of Four Novel Species in *Pleosporales* Associated with Coffee in Yunnan, China

**DOI:** 10.3390/jof8101113

**Published:** 2022-10-21

**Authors:** Li Lu, Samantha C. Karunarathna, Dong-Qin Dai, Yin-Ru Xiong, Nakarin Suwannarach, Steven L. Stephenson, Abdallah M. Elgorban, Salim Al-Rejaie, Ruvishika S. Jayawardena, Saowaluck Tibpromma

**Affiliations:** 1 Center for Yunnan Plateau Biological Resources Protection and Utilization, Yunnan Engineering Research College of Biological Re-Source and Food Engineering, Qujing Normal University, Qujing 655011, China; 2 Center of Excellence in Fungal Research, Mae Fah Luang University, Chiang Rai 57100, Thailand; 3 School of Science, Mae Fah Luang University, Chiang Rai 57100, Thailand; 4 Innovative Institute for Plant Health, Zhong Kai University, Guangzhou 510550, China; 5 Research Center of Microbial Diversity and Sustainable Utilization, Faculty of Science, Chiang Mai University, Chiang Mai 50200, Thailand; 6 Department of Biological Sciences, University of Arkansas, Fayetteville, AR 72701, USA; 7 Department of Botany and Microbiology, College of Science, King Saud University, Riyadh P.O. Box 145111, Saudi Arabia; 8 Department of Pharmacology & Toxicology, College of Pharmacy, King Saud University, Riyadh P.O. Box 145111, Saudi Arabia

**Keywords:** *Coffea*, *Deniquelata*, new species, new records, *Paraconiothyrium*, phylogeny, *Pseudocoleophoma*, taxonomy

## Abstract

In Yunnan Province, the coffee-growing regions are mainly distributed in Pu’er and Xishuangbanna. During the surveys of microfungi associated with coffee in Yunnan Province, seven taxa were isolated from coffee samples. Based on molecular phylogenetic analyses of combined ITS, LSU, SSU, *rpb*2, and *tef*1-α sequence data and morphological characteristics, four new species viz. *Deniquelata yunnanensis*, *Paraconiothyrium yunnanensis*, *Pseudocoleophoma puerensis*, and *Pse*. *yunnanensis*, and three new records viz. *Austropleospora keteleeriae*, *Montagnula thailandica*, and *Xenocamarosporium acaciae* in *Pleosporales* are introduced. In addition, *Paracamarosporium fungicola* was transferred back to *Paraconiothyrium* based on taxonomy and DNA sequences. Full descriptions, illustrations, and phylogenetic trees to show the placement of new and known taxa are provided. In addition, the morphological comparisons of new taxa with closely related taxa are given.

## 1. Introduction

The coffee genus *Coffea*, belonging to the botanical family *Rubiaceae*, has about 170 varieties [1], and one of the varieties, *Coffea arabica*, is the most popular coffee variety around the world [2]. Coffee, as the world’s second best-selling beverage and a food additive, sells all over the world, with many additional advantages such as refreshment, diuresis, invigorating the stomach, and stimulating appetite [3,4]. China’s total production of coffee ranks 12th place in the world, and the annual export volume of coffee beans reaches 82.7 thousand tons [5], making it the fourth largest coffee exporter in Asia after Vietnam, Indonesia, and India [5,6]. In 2020, the export and import of coffee in China were worth $145 million and $310 million, respectively [7]. Yunnan Province is the largest coffee capital in China, and the planting area and total yield account for 95–98% of the total in China [5,8].

Coffee is susceptible to microfungi during pre-harvest and post-harvest processing, especially pathogenic fungi, which can affect coffee tree growth, fruit yield, and quality [9,10]. The most recent coffee fungi review in 2022 counted about 966 coffee-associated microfungi records worldwide, belonging to 648 species, of which 295 are pathogenic fungi (the most common), while saprotrophic fungi are the least common (30 species) [10]. Several surveys of the literature also show that a few studies have been conducted on saprotrophic fungi associated with coffee [11]. Most of the studies focus on pathogens, as pathogenic fungal infections reduce coffee yield and quality, and thus affect farmers’ income and consumers’ health [12,13]. Some research has shown that saprotrophic fungus *Phialomyces macrosporus* isolated from leaf litter has the potential to be used in the management of coffee halo blight in seedlings [14]. Laborde et al. [15] demonstrated that volatile or non-volatile compounds produced by the saprotrophic fungus *Phialomyces macrosporus* can inhibit the growth, sporulation, and viability of the causative agent of the coffee brown eye-spot, *Cercospora coffeicola*. Therefore, saprotrophic fungi have the potential as a biological control agent to manage diseases. In China, a very few studies have been carried out on fungi associated with coffee. While most studies have been carried out on coffee pathogens, and only a few instances of saprotrophic fungi in coffee have been reported [3,8,10,16]. Yunnan is known as the province in China in which the most novel fungal are species reported [17]. In addition, coffee is one of the most important economic crops; thus, isolation and identification of coffee saprotrophic fungi based on morphology and multigene phylogeny are useful for future studies on the secondary metabolites of coffee fungi.

The largest fungal order, *Pleosporales*, was first proposed by Luttrell in 1955 and formally established by Barr in 1987, and belongs to the most diverse and qualified class, Dothideomycetes (Ascomycota) [18,19,20]. *Pleosporales* contains 91 families and 614 genera, and is distributed in various habitats worldwide (terrestrial and aquatic) [21,22]. Members of *Pleosporales* can be epiphytes, endophytes, or parasites of living leaves or stems. They can also be hyperparasites on fungi or insects, lichenized, or saprotrophic of dead plant stems, leaves, or bark [23,24]. *Pleosporales* are characterized by perithecioid ascomata, usually with a papillate apex, ostioles with or without periphyses, presence of cellular pseudoparaphyses, bitunicate asci, and ascospores of various shapes, pigmentations, and septations [24,25].

*Dictyosporiaceae* and *Didymosphaeriaceae* are two families of *Pleosporales*. *Dictyosporiaceae* was introduced with *Dictyosporium* as the type genus to accommodate a holomorphic group of Dothideomycetes by Boonmee et al. [26]. *Dictyosporium* contains saprotrophic fungi from decaying wood and plant debris in terrestrial and freshwater habitats [26,27]. *Didymosphaeriaceae* was introduced by Munk [28] and typified by *Didymosphaeria*. As a ubiquitous fungal family worldwide, *Didymosphaeriaceae* includes saprotrophic, endophytic, and pathogenic species associated with a wide variety of substrates [29].

Since Pu’er coffee and Xishuangbanna coffee are famous due to their strong but not bitter taste, fragrant but not hard smell, and slightly fruity flavor [30]. During our investigations of coffee saprotrophic fungi in Pu’er and Xishuangbanna in Yunnan Province, China, some interesting fungal taxa belonging to *Pleosporales* were found. The purposes of the current research are to isolate and identify the saprotrophic fungi associated with coffee trees based on morphological examination and multi-gene phylogeny, to provide full descriptions and photo plates of micromorphological characteristics, and to provide phylogenetic trees to show the placements of new and known taxa.

## 2. Materials and Methods

### 2.1. Specimen Collection, Morphological Study, and Isolation

Coffee branch samples with black fungal fruiting bodies visible to the naked eye were collected from coffee plantations in subtropical areas (Pu’er) and tropical areas (Xishuangbanna) in Yunnan Province in China. Each sample was placed in a separate plastic bag together with collection details such as collection date, collection site, and global positioning system (GPS) information, and then transported to the mycology laboratory at Qujing Normal University. Micro-morphological characteristics were observed and captured by differential interference contrast (DIC), using a Leica DM2500 compound microscope with a Leica DMC4500 camera. Measurements of microstructures were obtained by the Tarosoft (R) Image Frame Work program, while further processing was conducted in Adobe Photoshop CC 2018. Senanayake et al. [31] was followed for single spore isolation by using potato dextrose agar (PDA). Specimens were deposited at Zhongkai University of Agriculture and Engineering (ZHKU), while living cultures are maintained at the Zhongkai University of Agriculture and Engineering (ZHKUCC). Faces of fungi (FoF) numbers and Index Fungorum (IF) numbers were obtained as instructed in Jayasiri et al. [32] and Index Fungorum (2022) [33].

### 2.2. DNA Extraction, PCR Amplification and Sequencing

DNA extraction was carried out from two-week-old mycelium by using the Biospin Fungus Genomic DNA Extraction Kit-BSC14S1 (BioFlux^®^, Beijing, China), following the manufacturer’s instructions, and the methods of Lu et al. [3] were followed for the Polymerase Chain Reaction (PCR). The amplification and sequencing were carried out for five partial gene portions, the internal transcribed spacer (ITS) region was amplified with the primers ITS4 and ITS5 [34], the 18 s small subunit (SSU) region was amplified by primers NS1 and NS4 [34], the nuclear ribosomal 28 s large subunit (LSU) region with primers LR0R and LR5 [35], the partial RNA polymerase II subunit (*rpb*2) region with primers RPB2-5F and RPB2-7cR [36], and the partial translation elongation factor 1-alpha (*tef*1-α) gene with primers EF1-983F and 2218R [37]. The PCR mixture contains 8.5 µL of double-distilled water (ddH2O), 12.5 µL of 2 × Power Taq PCR MasterMix (mixture of EasyTaqTM DNA Polymerase, dNTPs, and optimized buffer, Beijing Bio Teke Corporation (Bio Teke), Beijing, China), 1 µL of each forward and reverse primers, and 2 µL of DNA. The conditions for PCR of ITS, LSU, SSU, and *tef*1-α genes constituted an initial denaturation step of 3 min at 94 °C, followed by 35 cycles of 45 s at 94 °C, 50 s at 55 °C, 60 s at 72 °C, and a final denaturation step of 10 min at 72 °C. For the *rpb*2 gene, the conditions constituted an initial denaturation step of 5 min at 95 °C, followed by 40 cycles of 60 s at 95 °C, 120 s at 55 °C, 90 s at 72 °C, and a final denaturation step of 10 min at 72 °C. The amplified PCR products were sent to Bioer Technology Co., Hangzhou, and Beijing Kinco Biotechnology Co., Kunming Branch, China. Generated sequences were deposited in GenBank and accession numbers were obtained (Table S1).

### 2.3. Sequence Alignment and Phylogenetic Analyses

Raw sequences, both reverse and forward, generated in this study were assembled with the Geneious program (9.1.2) [38]. The newly generated assembled sequences in this study were used for BLAST searches in GenBank [39]. The BLAST search results and sequences from the latest publications were used to obtain sequence data for the phylogenetic analyses. Single gene sequence alignments were made with the online program MAFFT v.7.110 [40]. They moved the uninformative gaps and ambiguous regions by trimAL v1.2 [41] and combined multigene sequencing by Sequence Matrix program (1.7.8) [42]. The fasta files have transferred the format in the AliView program [43], PHYLIP for maximum likelihood analysis (ML), and NEXUS for Bayesian analysis (BYPP).

Dissanayake et al. [44] was referenced for the phylogenetic analyses, considering both maximum likelihood and Bayesian methods. Maximum likelihood analysis was performed by RAxML-HPC v.8 on the online program CIPRES Science Gateway [45] with rapid bootstrap analysis, followed by 1000 bootstrap replicates, with GTRGAMMA substitution model. Bayesian analysis was performed by MrBayes v3.1.2, and the best models of evolution were estimated by MrModeltest 2.2 [46] and PAUP v. 4.0b10 [47]. The best-fit model was the GTR + I + G substitution model for each locus under the Akaike Information Criterion (AIC). Six simultaneous Markov Chains were run for 2 million generations, and trees were sampled at every 200th generation (resulting in 10,000 trees). Phylogenetic trees were visualized by FigTree v. 1.4.2 [48], the trees were edited in Microsoft Office PowerPoint 2020, and reliable bootstrap support values from ML and BYPP were inserted. All the obtained alignments and phylogenetic trees were deposited in Figshare [49] (https://doi.org/10.6084/m9.figshare.21260589 (accessed on 19 August 2022).) and TreeBASE (www.treebase.org (accessed on 19 August 2022), submission number 29752).

## 3. Results

### 3.1. Phylogenetic Analyses

Maximum likelihood phylogenetic analysis was conducted from combined SSU + LSU + ITS + *rpb*2 + *tef*1-α sequence data of 156 strains, of which 14 were newly sequenced strains, while the other 142 strains were obtained from BLAST search (NCBI) and recent papers [50,51,52,53,54]. Our phylogenetic trees show similar topologies to the recent published papers [50,51,52,53,54].

**Figure 1:**
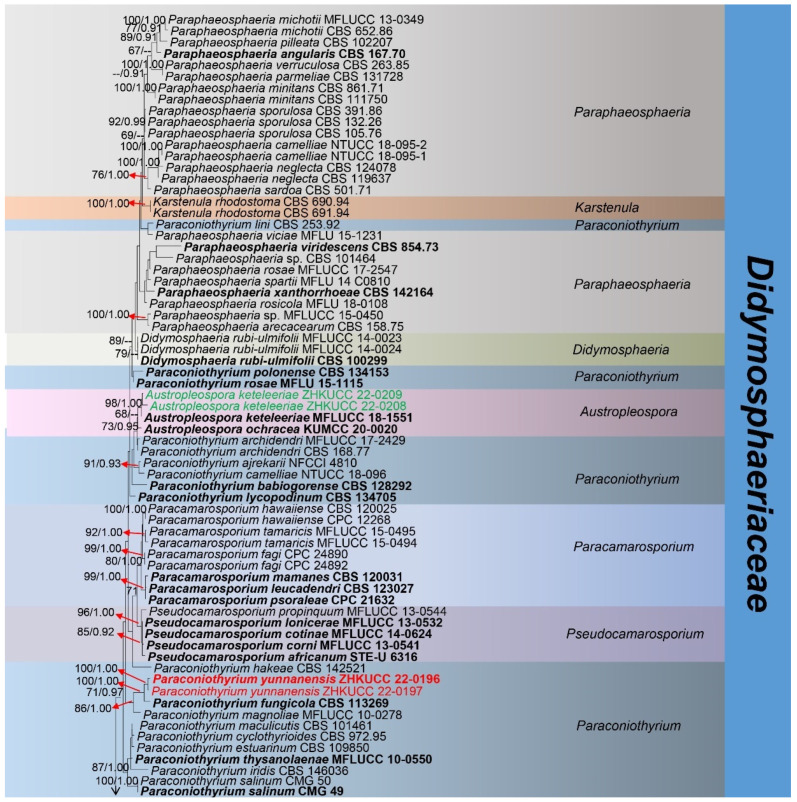

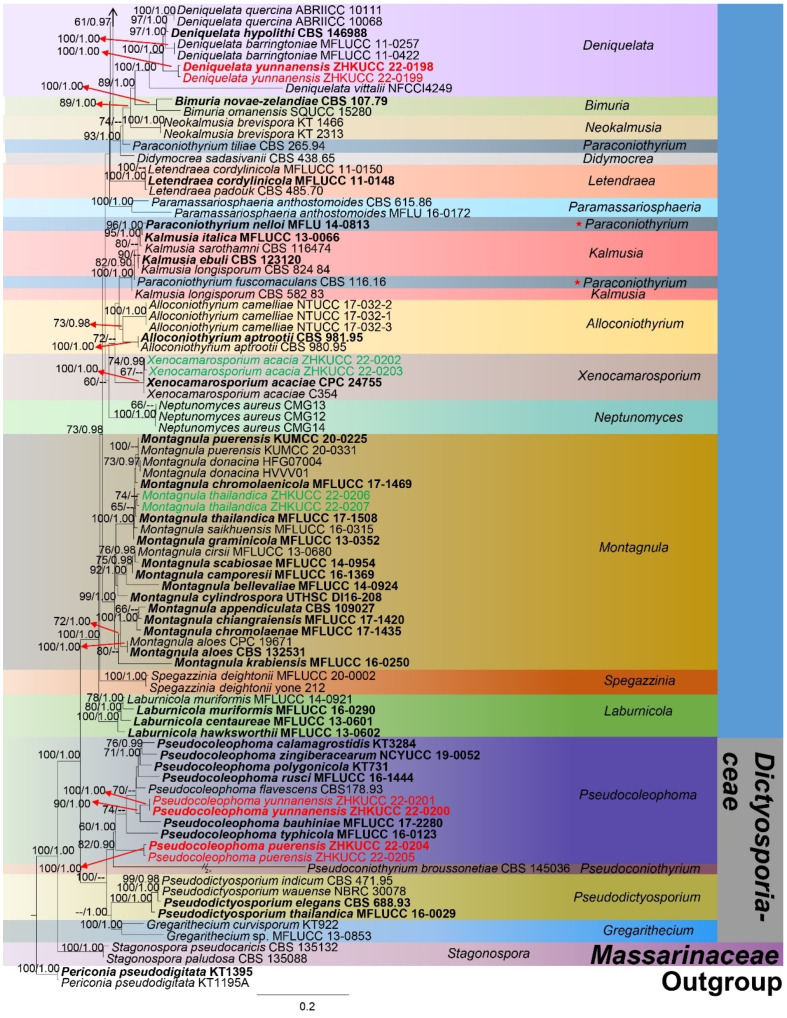
The best-scoring RAxML tree was constructed from a concatenated SSU, LSU, ITS, *rpb*2, and *tef*1-α dataset. The tree is rooted with *Periconia pseudodigitata* (KT1395, KT1195A). Nodes were annotated if the maximum likelihood bootstrap support value was ≥ 60% (ML, left) or if the Bayesian posterior probability was ≥ 0.90 (BYPP, right). The newly described species are in red, new records are in green, and type strains are in bold. Red stars are used to indicate the two species that have uncertain placements and are discussed in the discussion.

In each gene alignment, the missing data was calculated as 8.9% in the ITS gene, 7% in the LSU gene, 34% in the SSU gene, 65% in the *tef*1-α gene and 90% in the *rpb*2 gene. *Periconia pseudodigitata* strains KT1395 and KT1195A were selected as the outgroup taxa. The 156 strains comprised 4773 characters (SSU = 1–1098 bp, LSU = 1099–2018 bp, ITS = 2019–2677 bp, *rpb*2 = 2678–3776 bp, *tef*1-α = 3777–4773 bp) after alignment. The phylogenic tree from the RAxML analysis had a similar topology to the Bayesian analysis. The RAxML analysis of the combined dataset yielded the best-scoring tree (Figure 1) with a final ML optimization likelihood value of −36,512.450654. Alignment had 2112 distinct alignment patterns, with 50.81% gaps and completely undetermined characters. The estimated base frequencies were as follows: A = 0.242121, C = 0.244904, G = 0.272113, T = 0.240862; substitution rates were AC = 1.320338, AG = 2.546543, AT = 1.5403704, CG = 0.907448, CT = 6.221253, GT = 1.000000; gamma distribution shape parameter was α = 0.237001.

### 3.2. Taxonomy

***Dictyosporiaceae***  Boonmee and K.D. Hyde, Fungal Diversity 80: 462 (2016).

***Pseudocoleophoma***  Kaz. Tanaka and K. Hiray., Studies in Mycology 82: 89 (2015).

**Notes**: *Pseudocoleophoma* (*Pse*.) was introduced by Tanaka et al. [55] and was typified by *Pse. calamagrostidis* based on the pycnidial asexual morph, which differs from *Coleophoma* [55]. Most of the *Pseudocoleophoma* species were reported as asexual morphs [55,56], but only three species have been reported for both sexual and asexual morphs viz. *Pse. bauhiniae*, *Pse. calamagrostidis*, and *Pse. polygonicola* [55,57]. Sexual morph is characterized by ostiolar ascomata, brown and polygonal to rectangular cells of peridium, and numerous pseudoparaphyses, which are cylindrical to clavate and fissitunicate asci, fusiform, and septate ascospores, with an apparent sheath [55,57]. Asexual morph is characterized by pycnidial and subglobose conidiomata, phialidic and doliiform conidiogenous cells, and cylindrical or oblong, hyaline, aseptate, smooth-walled conidia [50].

In 2022, eight epithets (seven species) isolated as saprotrophic on different hosts and substrates from both terrestrial and freshwater habitats are listed in Index Fungorum (2022) [55,56,57,58,59,60]. *Pseudocoleophoma clematidis* was transferred to *Pseudocyclothyriella* by phylogenetic status and morphological distinctiveness [55]. Herein, we introduce two new species in *Pseudocoleophoma*, and this is the first report of *Pseudocoleophoma* from coffee.

**Figure 2:**
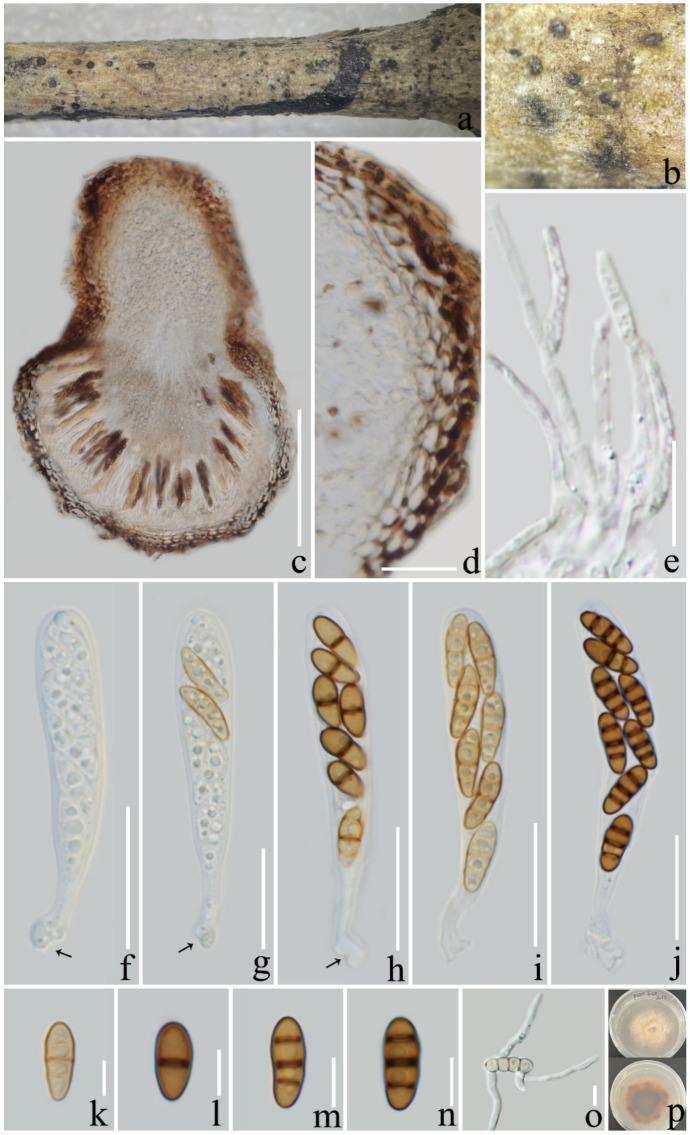
*Pseudocoleophoma puerensis* (ZHKU 22-0118). (**a**,**b**) Ascomata on a decayed branch of *Coffea arabica*; (**c**) section of an ascoma; (**d**) peridium at side; (**e**) pseudoparaphyses; (**f**–**j**) immature and mature asci (arrows indicate the club-like pedicel); (**k**–**n**) ascospores; (**o**) germinated ascospore; (**p**) culture on PDA from above and reverse (60 days). Scale bars: (**c**,**e**) = 100 µm; (**f**–**j**) = 20 µm; (**k**–**n**) = 5 µm; (**o**) = 10 µm.

***Pseudocoleophoma puerensis*** L. Lu and Tibpromma, sp. nov. (Figure 2)

Index Fungorum number: IF 559421; Faces of Fungi number: FoF 12761

**Etymology**:  Name reflects the location “Pu’er” City in Yunnan Province, where the holotype was collected.

**Holotype**:  ZHKU 22-0118.

*Saprotrophic* on decaying branch of *Coffea arabica* var. *catimor*. *Ascomata:* 150–300 × 170–220 μm ($\bar{x}$ = 197 × 199 μm, *n* = 15) (includes ostiolar neck), solitary, immersed to erumpent, brown to black, globose or subglobose. *Peridium:* 14–20 μm wide ($\bar{x}$ = 17 μm, *n* = 20), thin, wall composed of 2–4 layers *textura angularis* cells, brown to dark brown. *Hermathecium:* 1.5–3 µm wide ($\bar{x}$ = 2.3 μm, *n* = 20) septate, branched, numerous, hyaline, pseudoparaphyses. *Asci:* 50–70 × 7–11 μm ($\bar{x}$ = 57 × 8.5 μm, *n* = 20), 8-spored, fissitunicate, cylindrical, rounded at the apex, with a shallow ocular chamber, long-stalked with club-like pedicel. *Ascospores:* 10–15 × 3.5–6 μm ($\bar{x}$ = 11.6 × 4, *n* = 20), uniseriate to biseriate, narrowly ellipsoid or oblong, 1–3 thick and dark eusepta, slightly constricted at the septa, straight or slightly curved, hyaline and yellowish brown when young, turning brown when mature, normally 4 guttulate, without sheath. **Asexual morph**: undetermined.

**Culture characteristics**: Colonies on PDA after two months attaining a diam of 40 mm at 25 °C, above side: pale yellow, circular, flatted, margin filamentous; reverse side: center brown, middle off yellowish brown and white at the margin.

**Material examined**: China, Yunnan Province, Pu’er City, on a decaying branch of *Coffea arabica* var. *catimor*, (100°56′9″ E, 22°40′28″ N, 1090.5 m), 16 September 2021, LiLu, Pu’er 3-2 (ZHKU 22-0118, holotype), living culture ZHKUCC 22-0204 = ZHKUCC 22-0205. GenBank number; ITS: OP297799, LSU: OP297769, SSU: OP297783; *tef*1-α: OP321568 (ZHKUCC 22-0204, ex-type); ITS: OP297800, LSU: OP297770, SSU: OP297784; *tef*1-α: OP321569 (ZHKUCC 22-0205).

**Notes**: In the phylogenetic tree constructed based on multigene data, our isolates clustered within *Pseudocoleophoma* and formed a distinct branch basal to *Pse. typhicola* (MFLUCC 16-0123), with fairly good statistical support (60% ML/1.00 BYPP, Figure 1). However, *Pse. typhicola* has only been reported as an asexual morph [56]. Based on nucleotide comparisons, our isolate (ZHKUCC 22-0204) differs from *Pse. typhicola* (MFLUCC 16-0123) by 63/533 bp (11%) in ITS, 18/669 bp (2.6%) in LSU, while it lacks the SSU and *tef*1-α genes. The morphology of our isolate is similar to *Pseudocoleophoma*, which was described by Tanaka et al. [55], although we noticed that the ascospores are 1–3 septate and brown, with no sheath in ascospores. Therefore, herein we introduce *Pse. puerensis* as a new species in *Pseudocoleophoma*.

**Figure 3:**
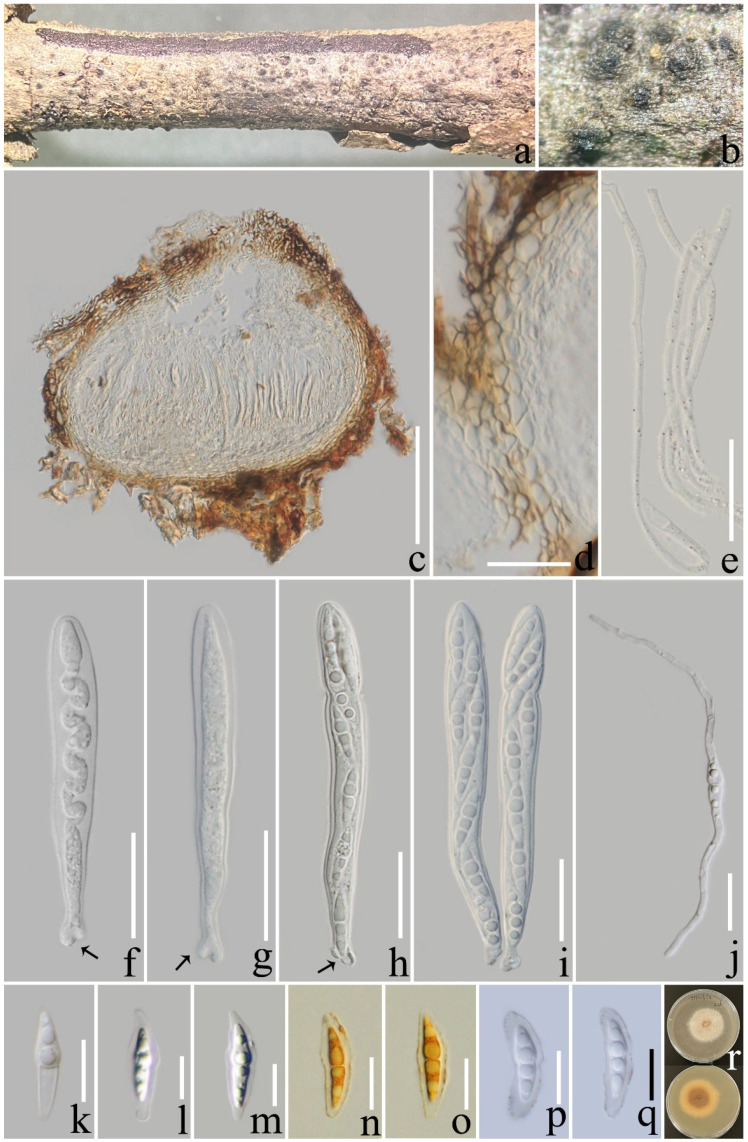
*Pseudocoleophoma yunnanensis* (ZHKU 22-0116). (**a**,**b**) Ascomata on a decayed branch of *Coffea* sp.; (**c**) section of an ascoma; (**d**) peridium at side; (**e**) pseudoparaphyses; (**f**–**i**) immature and mature asci (arrows indicate the club-shape pedicel); (**j**) germinated ascospore; (**k**–**m**) ascospores; (**n**,**o**) ascospore stained with Lugol’s iodine; (**p**,**q**) ascospore stained with Indian ink; (**r**) culture on PDA from above and reverse (60 days). Scale bars: (**c**) = 100 µm; (**d**–**j**) = 20 µm; (**k**–**q**) = 10 µm.

***Pseudocoleophoma yunnanensis*** L. Lu and Tibpromma, sp.nov. (Figure 3).

Index Fungorum number: IF 559422; Faces of Fungi number: FoF 12762.

**Etymology**:  Name reflects the location “Yunnan” Province, where the holotype was collected.

**Holotype**:  ZHKU 22-0116.

*Saprotrophic* on decaying branch of *Coffea* sp. **Sexual morph**: *Ascomata*: 160–280 × 200–280 µm ($\bar{x}$ = 216 × 243 µm; *n* = 15), semi-immersed to erumpent, solitary or scattered, coriaceous, subglobose to obpyriform, dark brown to black, with protruding ostiolar neck. *Peridium*: 22–27 µm wide ($\bar{x}$ = 23; *n* = 15), outer walls comprising 2–4 layers of *textura angularis* cells, hyaline to brown, inner walls hyaline, density, several layers of *textura angularis* cells. *Hamathecium*: 1.5–3 µm wide ($\bar{x}$ = 2.1 μm; *n* = 15), hyaline, septate, branched, pseudoparaphyses numerous. *Asci*: 65–90 × 8–11 µm ($\bar{x}$ = 76 × 9 µm; *n* = 20), 8-spored, fissitunicate, clavate to cylindrical, some slightly curved, constricted at the upper part when mature, with an ocular chamber, short-stalked with club-shape pedicel. *Ascospores*: 16–26 × 4–8 ($\bar{x}$ = 21 × 6 µm; *n* = 20), uniseriate to biseriate, hyaline, fusiform, straight to slightly curved, 1-septate, 4 guttulate, smooth-walled, with a distinct sheath. **Asexual morph**: undetermined.

**Culture characteristics**: Ascospores germinated on PDA within 12 h; colonies on PDA after two months attaining a diam of 40 mm at 25 °C, slightly raised, fluffy, white, circular, margin filamentous, reverse pale yellow to light brown from edge to center.

**Material examined**: China, Yunnan Province, Xishuangbanna, on a decaying branch of *Coffea* sp., (1672 m), 12 September 2021, LiLu, JHMH 15 (ZHKU 22-0116, holotype), living culture ZHKUCC 22-0200 = ZHKUCC 22-0201. GenBank number: ITS: OP297795, LSU: OP297765, SSU: OP297779, *tef*1-α: OP321564 (ZHKUCC 22-0200, ex-type); ITS: OP297796, LSU: OP297766, SSU: OP297780, *tef*1-α: OP321565 (ZHKUCC 22-0201).

**Notes**: In the phylogenetic tree, our species, *Pseudocoleophoma yunnanensis*, formed a well-separated clade clustered with *Pse. bauhiniae* (MFLUCC 17-2280) with high statistical support (90% ML/1.00 BYPP, Figure 1). Based on nucleotide comparisons, our isolate (ZHKUCC 22-0200) is different from *Pse. bauhiniae* (MFLUCC 17-2280) by 13/489 bp (2.6%) in ITS, 3/833 bp (0.4%) in LSU, 9/1069 bp (0.8%) in SSU, and 73/878bp (8.3%) in *tef*1-α. In addition, morphological features of the sexual morph *Pse. yunnanensis* can be distinguished by having constricted asci and the presence of a distinct sheath of ascospores with *Pse. bauhiniae* and other species in *Pseudocoleophoma* [51,55,58]. Therefore, we introduce *Pse. yunnanensis* as a new species that was isolated from coffee.

***Didymosphaeriaceae***  Munk, Dansk botanisk Arkiv 15 (2): 128 (1953).

***Austropleospora***  R.G. Shivas and L. Morin, Fungal Diversity 40 (1): 70 (2010).

**Notes**: *Austropleospora* (*A*.) was introduced by Morin et al. [61], with *A. osteospermi* as the type species. This was isolated as a pathogen with both sexual and asexual morphs from stems of *Chrysanthemoides monilifera* subsp. *Rotundata*, with dieback symptoms in Australia, but its pathogenicity has not been accurately confirmed [61]. The sexual morphs are characterized by immersed and ostiolate ascomata, filamentous and septate pseudoparaphyses, bitunicate, clavate to cylindrical asci, and ellipsoid, yellowish-brown ascospores. The asexual morph has coelomycetous, pycnidial, globose conidiomata, brown to reddish-brown conidiomata walls, and yellowish-brown globose to obovate conidia [51,62,63]. Species of this genus have been reported as pathogenic or saprotrophic in Australia, China, and Thailand [56,60,62,63]. *Austropleospora* contains four epithets (three species) in Index Fungorum (2022) viz. *A. archidendri*, *A. keteleeriae*, *A. ochracea*, and *A. osteospermi*, while *A. archidendri* has been synonymized under *Paraconiothyrium* [62]. Thus, we introduce the new host record *Austropleospora keteleeriae* from coffee.

**Figure 4:**
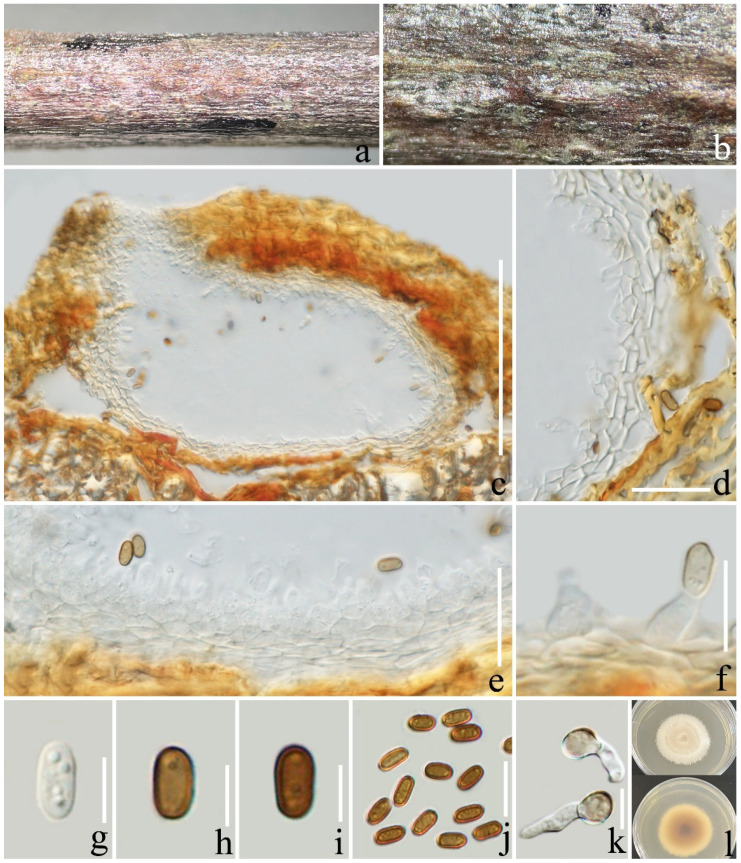
*Austropleosporaketeleeriae* (ZHKU 22-0120). (**a**,**b**) Appearance of the conidiomata on decaying branch of *Coffea* *arabica* var. *catimor*; (**c**) cross sections of a conidioma; (**d**) pycnidial wall; (**e**,**f**) conidia attached to conidiogenous cells; (**g**–**j**) conidia; (**k**) germinated conidium; (**l**) culture on PDA from above and reverse (60 days); scale bars: (**c**) = 100 µm; (**d**,**e**) = 20 µm; (**f**,**k**) = 10 µm; (**g**–**j**) = 5 µm.

***Austropleospora keteleeriae***  Jayasiri, E.B.G. Jones and K.D. Hyde, Mycosphere 10 (1): 65 (2019) (Figure 4)

Index Fungorum number: IF 555541; Faces of Fungi number: FoF 05244.

*Saprotrophic* on a decaying branch of *Coffea arabica* var. *catimor*. **Sexual morph**: undetermined. **Asexual morph**: Coelomycetous. *Conidiomata*: 70–150 × 150–250 μm ($\bar{x}$ = 104 × 209 μm, *n* = 15), pycnidial, immersed, solitary, globose to obpyriform, or irregular, short ostiolate after maturity. *Conidiomata wall*: 12–17 ($\bar{x}$ = 14.5 μm, *n* = 20), composed of 2–3 layers, inner wall composed of 1–2 layers, hyaline cells of *textura angularis*. *Conidiophores*: reduced to conidiogenous cells. *Conidiogenous cells*: 3–6 × 2–4 μm ($\bar{x}$ = 4.3 × 2.5 µm, *n* = 20), enteroblastic, phialidic, globose to doliiform, hyaline, smooth-walled, lining the inner wall layer of the pycnidium. *Conidia*: 4–7 × 2.5–4.5 μm ($\bar{x}$ = 6 × 3.5 μm, *n* = 30), solitary, the color changes with different ages, hyaline when young, brown to dark-brown; when mature, subglobose to obovate, one-celled, thick and smooth-walled, aseptate, with small oil droplets.

**Culture characteristics**: Conidia germinated within 12 h on PDA, growing on PDA reaching around 40 mm after two months at room temperature (25 °C). Above: white, circular, surface flocculent, with a significant amount of aerial mycelia. Reverse: the color gradually becomes darker from the edge to the center, white, yellowish to light brown.

**Material examined**: China, Yunnan Province, Pu’er City, on a decaying branch of *Coffea arabica* var. *catimor*, (100°56′9″ E, 22°40′28″ N, 1090.5 m), 16 September 2021, LiLu, Pu’er 3-9 (ZHKU 22-0120), living culture ZHKUCC 22-0208 = ZHKUCC 22-0209. GenBank number; ITS: OP297801, LSU: OP297771, SSU: OP297785, *tef*1-α: OP321570 (ZHKUCC 22-0208); ITS: OP297802, LSU: OP297772, SSU: OP297786, *tef*1-α: OP321571 (ZHKUCC 22-0209).

**Notes**: *Austropleospora keteleeriae* was isolated as saprotrophic on a decaying cone of *Keteleeria fortunei* from China [58]. Our new isolates form a sister clade to *A. keteleeriae* in the phylogenetic analyses, with high statistical support (98 ML/1.00 BYPP, Figure 1). The morphological characteristics of the new isolates fit with *A. keteleeriae* by having conidiomata consisting of textura angularis cells, phialidic, enteroblastic, hyaline, and smooth conidiogenous cells, and globose to obovate, one-celled, thick and smooth-walled, hyaline to brown conidia [58]. While ITS blast results showed that our strain is 99% similar to *A. archidendri* (*Paraconiothyrium archidendri*) (MZ855427), the results of the LSU blast showed that it is 100% similar to *Paraconiothyrium* sp. (JX496165), the results of the SSU blast showed that it is 99.9% similar to *Austropleospora* sp. (MT808321), and the *tef*1-α blast showed that it is 99.8% similar to *A. keteleeriae* (MK360045). Therefore, we introduce *A. keteleeriae* as a new host record from a decaying branch of *Coffea arabica* var. *catimor* in China, based on morphology and phylogeny.

***Deniquelata***  Ariyaw. and K.D. Hyde, Phytotaxa 105 (1): 15 (2013).

**Notes**: *Deniquelata* (*D*.) was introduced by Ariyawansa et al. [52], with *D. barringtoniae* as the type species, which was isolated as a pathogen, causing leaf spots of *Barringtonia asiatica* (*Lecythidaceae*). The sexual morph of *Deniquelata* is characterized by immersed ascomata, with an ostiolar, textura angularis cell peridium, bitunicate, clavate to broadly-clavate with short furcate pedicel asci and muriform ascospores, three transverse septa and 1–2 vertical septa, without a sheath [52,64]. The asexual morph is characterized by branched and septate mycelium, conidiophores reduced to conidiogenous cells, pale brown and subcylindrical conidiogenous, subglobose to pyriform and guttulate conidia, with 1–3 transverse and 0–2 vertical septa [65]. Species of this genus have been reported as pathogens, saprophytes, and endophytes [52,64,65,66]. Currently, the genus *Deniquelata* contains four species in the Index Fungorum (2022) viz. *D. barringtoniae*, *D. hypolithi*, *D. quercina*, and *D. vittalii*. In this study, *Deniquelata yunnanensis* is introduced from coffee in Yunnan Province, China, and this is the first report of the genus *Deniquelata* from coffee.

**Figure 5:**
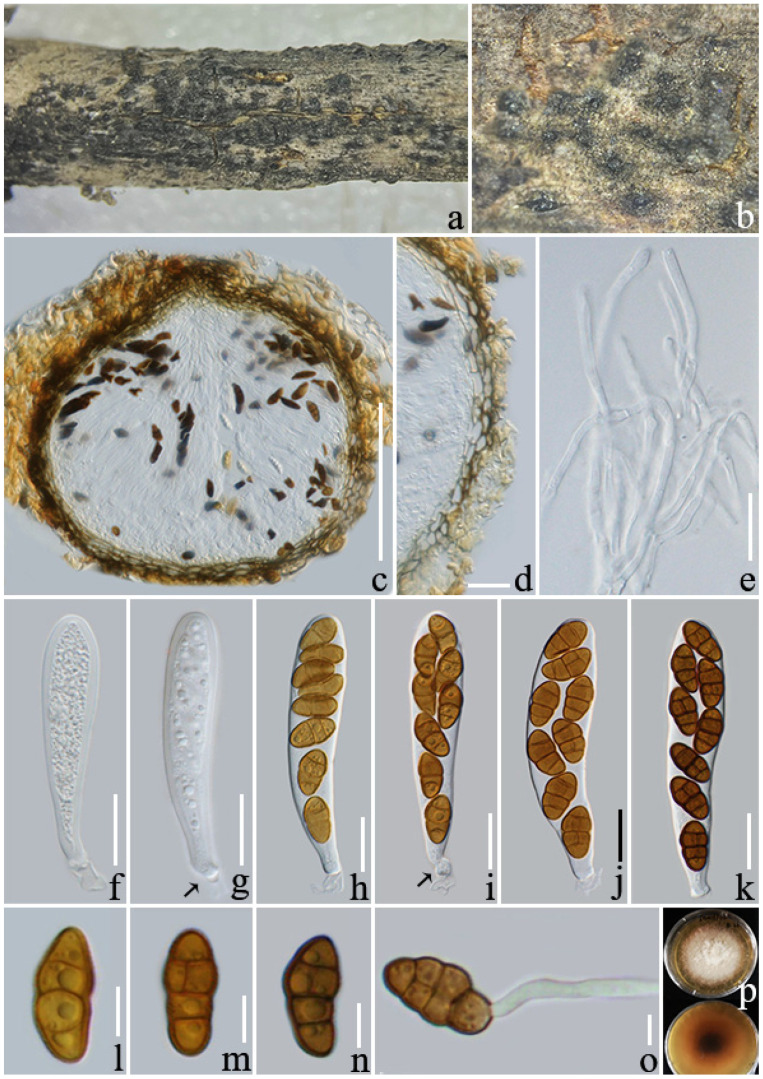
*Deniquelata yunnanensis* (ZHKU 22-0115). (**a**,**b**) Ascomata on a decaying branch of *Coffea* sp.; (**c**) vertical section of an ascoma; (**d**) peridium; (**e**) pseudoparaphyses; (**f**–**k**) asci (arrows indicate the short furcate pedicel); (**l**–**n**) ascospores; (**o**) germinated ascospore; (**p**) culture on PDA from above and reverse (30 days). Scale bars: (**c**) = 100 µm; (**d**–**k**) = 15 µm; (**l**–**o**) = 5 µm.

***Deniquelata yunnanensis*** L. Lu and Tibpromma, sp. nov. (Figure 5),

Index Fungorum number: IF 559423; Faces of Fungi number: FoF 12763.

**Etymology**:  Name reflects the location, “Yunnan” Province, where the holotype was collected.

**Holotype**:  ZHKU 22-0115.

*Saprotrophic* on decaying branch of *Coffea* sp. **Sexual morph**: *Ascomata*: 140–240 × 170 −240 μm ($\bar{x}$ = 187 × 209 μm, *n* = 20), semi-immersed, aggregated to solitary, globose to subglobose, dark brown to black, with ostiole and apex papillate to depressed. *Peridium:* 12−17 µm wide ($\bar{x}$ = 14, n = 15), composed of two walls, outer wall light brown to brown, comprising 2–3 layers, cells of *textura angularis*, fused with the host cells, inner wall thin, hyaline cell. *Hamathecium:* composed of dense, 1.5−3 µm wide ($\bar{x}$ = 2, *n* = 20), branched, hyaline, septate pseudoparaphyses, surrounding the numerous asci and enclosed in a gelatinous matrix. *Asci:* 50–85 × 10–17 μm ($\bar{x}$ = 68 × 13 μm, *n* = 30), 8-spored, bitunicate, fissitunicate, clavate, with a short furcate pedicel. *Ascospores:* 11–16 × 4–8 μm ($\bar{x}$ = 14 × 6.6 μm, *n* = 30), 2-seriated ascospores, partially overlapping, muriform, hyaline when young, yellow to brown at maturity and with distinct guttulate, ellipsoidal to oblong, with 0–2 longitudinal septate in each cell, 1–3 transverse septate, constricted at the septa, slightly curved to straight, apically conical to elliptical, without a sheath. **Asexual morph**: undetermined.

**Culture characteristics**: Ascospores germinated within 12 h on PDA, growing on PDA and reaching around 50 mm after one month at room temperature (25 °C). The surface is white to light grey, circular, cottony and fluffy, slightly raised in the middle, margin filamentous, reverse yellowish to light brown.

**Material examined**: China, Yunnan Province, Xishuangbanna, on a decaying branch of *Coffea* sp., (1672 m), 12 September 2021, LiLu, JHMH 10 (ZHKU 22-0115, holotype), living culture ZHKUCC 22-0198 = ZHKUCC 22-0199. GenBank number; ITS: OP297803, LSU: OP297773, SSU: OP297787, *tef*1-α: OP321572, *rpb*2: OP321562 (ZHKUCC 22-0198, ex-type); ITS: OP297804, LSU: OP297774, SSU: OP297788, *tef*1-α: OP321573, *rpb*2: OP321563 (ZHKUCC 22-0199).

**Notes**: In the phylogenetic analyses, *Deniquelata yunnanensis* formed a distinct sister clade to *Deniquelata* with strong statistical support (100 ML/1.00 BYPP, Figure 1). In the NCBI blast results of sequences, ITS was 92.8% similar to *D. barringtoniae* (MH141242), LSU/*tef*1-α/*rpb*2 was similar to *D. hypolithi* (NG_076735, 97.8%), (MZ078250, 97%), and (MZ078201, 94%), respectively, while SSU highly overlapped with *Deniquelata* sp. (MH316155) at 99%. Morphologically, our strains well fit with the generic characteristics of *Deniquelata* [52,64]. An asexual morph of *D. hypolithi* was reported [65], while the sexual morph of *D. yunnanensis* can be distinguished from *D. barringtoniae* by the color of the ascospores, which are yellow to brown at maturity and have a distinct guttulate as well as 1–3 transverse septa and 0–2 longitudinal septa in each ascospore, while ascospores in *D. barringtoniae* are reddish-brown, with three transverse septa and 1−2 vertical septa in each ascospore [63]. Both of the phylogenetic analyses and morphological characteristics supported our species as a distinct new species in *Deniquelata*.

***Montagnula***  Berl., Icones Fungorum. Pyrenomycetes 2: 68 (1896).

**Notes**: *Montagnula* (*M*.) was introduced in *Montagnulaceae* by Berlese in 1896 to accommodate *M. gigantean* and *M. infernalis* (the type species) [67,68]. The sexual morph of the genus is characterized by globose or spherical and immersed ascomata with clypeus, claviform asci, fusoid, or ellipsoid ascospores, as well as with transverse septa and one or more longitudinal septa [55,69], while the asexual morph remains undetermined [70]. According to the multi-gene phylogeny inferred from the combined dataset, *Didymosphaeriaceae* incorporates members of *Montagnulaceae*, so the genus *Montagnula* was moved to *Didymosphaeriaceae* [70]. The genus comprises saprotrophic fungi growing on dead wood, branches, stems, bark, and leaves, which play an important role [70,71,72,73]. A total of 47 epithets (43 species) are listed in Index Fungorum (2022). The species of *Montagnula* are distributed over 29 countries and 65 host species [54,74]. In this paper, we introduce a new host and country record in *Montagnula* from coffee in China.

**Figure 6:**
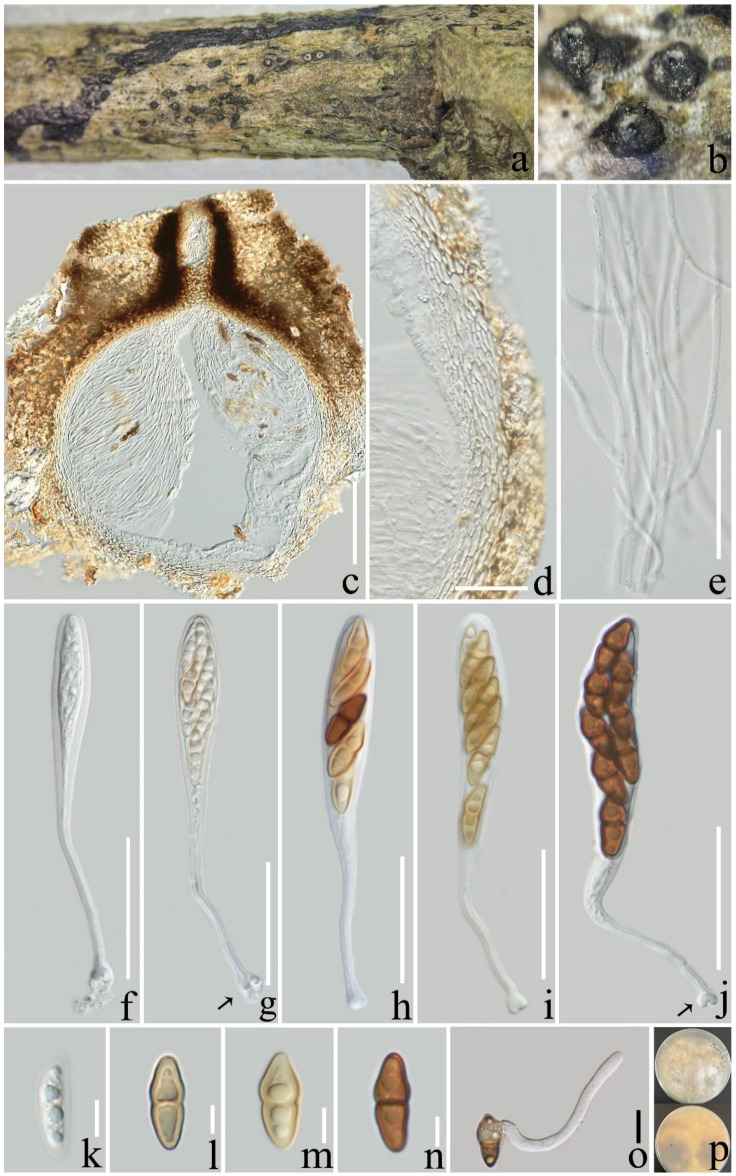
*Montagnula thailandica* (ZHKU 22-0119). (**a**,**b**) Ascomata on a decaying branch of *Coffea* *arabica*; (**c**) vertical section of an ascoma; (**d**) peridium; (**e**) pseudoparaphyses; (**f**–**j**) asci (arrows indicate the club-shape pedicel); (**k**–**n**) ascospores; (**o**) germinated ascospore; (**p**) culture on PDA from above and reverse (30 days). Scale bars: (**c**) = 100 µm; (**d**–**j**) = 30 µm; (**k**–**n**) = 5 µm; (**o**) = 10 µm.

***Montagnula thailandica***  Mapook and K.D. Hyde, Fungal Diversity 101: 35 (2020) (Figure 6)

Index Fungorum number: IF 557299; Faces of Fungi number: FoF 07792.

*Saprotrophic* on a decaying branch of *Coffea arabica* var. *catimor*. **Sexual morph**: *Ascomata:* 300–500 × 280–350 µm ($\bar{x}$ = 26 µm, *n* = 10), semi-immersed to erumpent, brown to black, spherical to obpyriform, solitary or scattered, with papillate ostiole. *Peridium:* 20–30 µm wide ($\bar{x}$ = 377 × 313 µm, *n* = 10), comprising several layers of thin-walled, hyaline to brown cells of *textura angularis*. *Hamathecium:* 1.5–2.5 µm wide, hyaline, cellular, branched, septate, numerous pseudoparaphyses. *Asci:* 80–110 × 9–13 µm ($\bar{x}$ = 93 × 11 µm, *n* = 25), 6–8-spored, bitunicate, elongate-clavate, long-stalked with club-shape pedicel, slightly curved. *Ascospores:* 12–16 × 5.5–6.5 µm ($\bar{x}$ = 14 × 5.8 µm, *n* = 25), overlapping 1–2-seriate, hyaline to yellowish-brown when immature, brown to reddish-brown when mature, broadly fusiform to ellipsoid, 1-septate, constricted at the septum, slightly wider upper cell and tapering towards ends, straight or slightly curved, with 2–4 guttulate, without terminal appendages or a sheath. **Asexual morph**: undetermined.

**Culture characteristics**: Ascospores germinated on PDA within 24 h at room temperature (25 °C). One month after growing on PDA, it reached 60 mm. The obverse mycelium is fluffy, slightly raised, white to light brown, circular, filamentous at the margin; the reverse is yellowish to dark brown from edge to center.

**Material examined**: China, Yunnan Province, Pu’er City, on a decaying branch of *Coffea arabica* var. *catimor*, (100°56′9″ E, 22°40′28″ N, 1090.5 m), 16 September 2021, LiLu, Pu’er 3-4 (ZHKU 22-0119), ZHKUCC 22-0206 = ZHKUCC 22-0207. GenBank number; ITS: OP297807, LSU: OP297777, SSU: OP297791, *tef*1-α: OP321576 (ZHKUCC 22-0206); ITS: OP297808, LSU: OP297778, SSU: OP297792, *tef*1-α: OP321577 (ZHKUCC 22-0207).

**Notes**: *Montagnula thailandica* was introduced as a new species from dead stems of *Chromolaena odorata* from Thailand by Mapook et al. [73], based on the morphology of the sexual morph and the phylogenetic analyses. In this paper, our new isolates clustered with the ex-type strain of *M. thailandica* (MFLUCC 17-1508) with fairly good bootstrap support (ML/BI = 74/-). The morphology of the new isolate (ZHKU 22-0119) is very similar to the holotype *M. thailandica* (MFLU 20-0325) in size and color of asci and ascospores [73]. Based on blast results, ITS and *tef*1-α are 99.6% similar to *M. thailandica* (MFLUCC 17-1508), and LSU and SSU are 100% similar to *M. thailandica* (MFLUCC 17-1508). Therefore, the new isolate (ZHKU 22-0119) is identified as *M. thailandica*, and this is a new host and new country record of *M. thailandica*, isolated from a decaying branch of *Coffea arabica* in China.

***Paraconiothyrium***  Verkley, Studies in Mycology 50 (2): 327 (2004).

**Notes**: *Paraconiothyrium* (*Paraco*.) was proposed to accommodate four new species, *Paraco. estuarinum*, *Paraco. brasiliense*, *Paraco. cyclothyrioides*, and *Paraco. fungicola* by Verkley et al. [75]. The genus is reported as a phytopathogen, saprophyte, and endophyte in a wide range of hosts and substrates worldwide [69,75,76]. The asexual morph characteristics of *Paraconiothyrium* are eustromatic conidiomata, phialidic conidiogenous cells and aseptate, sometimes 1-septate, thin-walled, smooth or minutely warted, and hyaline to brown conidia [75]. Sexual morph characteristics are globose or subglobose ascomata, clavate or cylindrical asci, and fusiform to ellipsoidal ascospores [76]. The genus comprises 29 epithets (21 species) in the Index Fungorum (2022), but some species have already been moved to other genera. The taxonomic affiliation of the *Paraconiothyrium* species is still confusing, with contrasting differences at the phylogenetic and morphological levels [77]. In our study, we introduce a new species of *Paraconiothyrium* from coffee.

**Figure 7:**
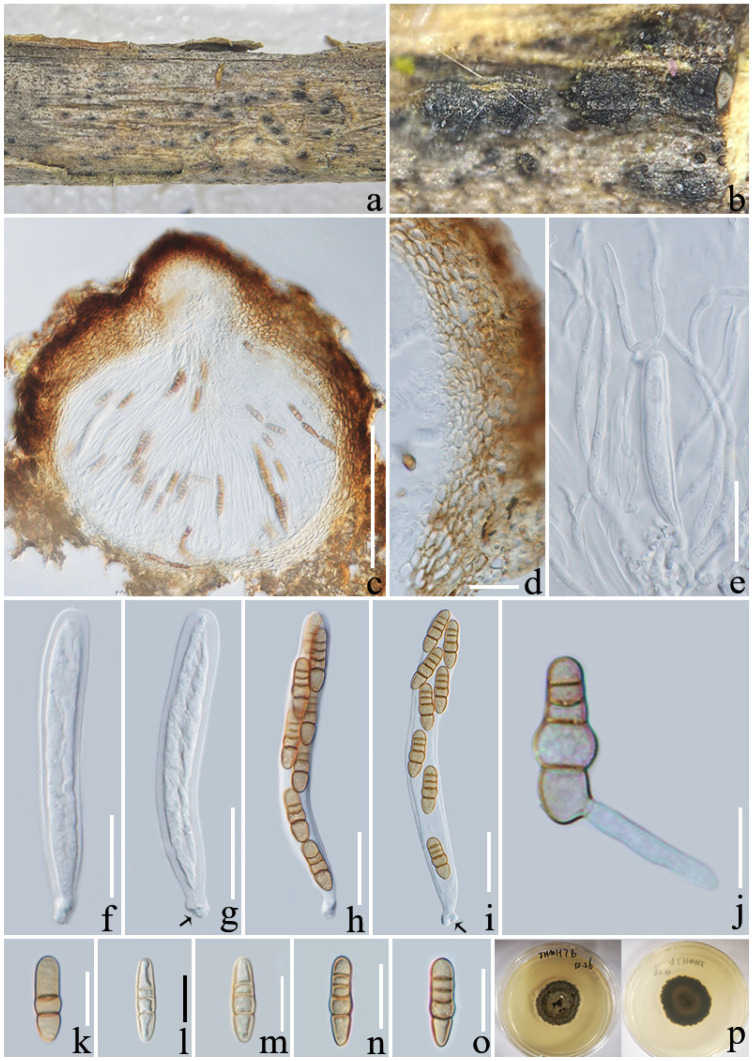
*Paraconiothyrium yunnanensis* (ZHKU 22-0114). (**a**,**b**) Ascomata on a decaying branch of *Coffea* sp.; (**c**) vertical section of an ascoma; (**d**) peridium; (**e**) pseudoparaphyses; (**f**–**i**) asci (arrows indicate the club-like pedicel); (**j**) germinated ascospore; (**k**–**o**) ascospores; (**p**) culture on PDA from above and reverse (30 days). Scale bars: (**c**) = 100 µm; (**d**–**i**) = 15 µm; (**j**–**o**) = 10 µm.

***Paraconiothyrium yunnanensis*** L.  Lu and Tibpromma, sp. nov. (Figure 7),

Index Fungorum number: IF 559424; Faces of Fungi number: FoF 12764.

**Etymology**:  Name reflects the location, “Yunnan” Province, where the holotype was collected.

**Holotype**:  ZHKU 22-0114.

*Saprotrophic* on a decaying branch of *Coffea* sp. **Sexual morph**: *Ascomata:* 150–250 × 160–260 μm ($\bar{x}$ = 193 × 214 μm, *n* = 20), solitary, scattered, immersed to semi-immersed, brown to black, globose or subglobose, coriaceous, small papilla, with small ostiole, ostiolar canal lined without hyaline periphyses. *Peridium:* 16–28μm ($\bar{x}$ = 22 μm, *n* = 20) wide, thick, composed of 4–6 layers of *textura prismatica* cells, yellowish to brown. *Hamathecium:* 1.5–3 µm wide ($\bar{x}$ = 2.2 μm, n = 20), hyaline, branched and septate, numerous pseudoparaphyses. *Asci:* 60–110 × 8–11 μm ($\bar{x}$ = 79 × 9.3 μm, *n* = 30), 8-spored, bitunicate, fissitunicate, oblong or cylindrical, short-stalked, some with club-like pedicels, slightly curved. *Ascospores:* 15–18 × 4–5 μm ($\bar{x}$ = 16 × 4.6, *n* = 30), biseriate or partially overlapping, fusiform or ellipsoidal, yellowish to light brown, 2–4-septate, smooth-walled, penultimate cell enlarged, without a sheath or guttulate. **Asexual morph**: undetermined.

**Culture characteristics**: Ascospores germinated within 12 h on PDA, growing on PDA and reaching around 40 mm after one month at room temperature (25 °C). Colonies were circular, filiform, dark green, with the mycelium raised and a lot of aerial hyphae in the edges; the center of the reverse was brown with dark green edges.

**Material examined**: China, Yunnan Province, Xishuangbanna, on a decaying branch of *Coffea* sp., (1672 m), 12 September 2021, LiLu, JHMH 7 (ZHKU 22-0114, holotype), living culture ZHKUCC 22-0196 = ZHKUCC 22-0197. GenBank number; ITS: OP297797, LSU: OP297767, SSU: OP297781, *tef*1-α: OP321566, *rpb*2: OP321560 (ZHKUCC 22-0196, ex-type); ITS: OP297798, LSU: OP297768, SSU: OP297782, *tef*1-α: OP321567, *rpb*2: OP321561 (ZHKUCC 22-0197).

**Notes**: Phylogenetic analyses show that *Paraconiothyrium yunnanensis* is well-separated from *Paraco. fungicola*, with 100% ML, 1.00 BYPP statistical support (Figure 1). Based on BLAST search results of sequence data, ITS and LSU are closely related to *Paraco. fungicola*, with similarity rates of 98.9% (MK619287) and 99.8% (JX496133). SSU is 99.8% similar to *Paraco. variabile* (KM096136), *rpb*2 is 88% (MT473955) similar to *Paraconiothyrium* sp., and *tef*1-α is 97% (LT797134) similar to *Paraco. cyclothyrioides*. In terms of morphological characteristics, our new species is similar to *Paraco. magnoliae* in that it has ellipsoidal, yellowish to light brown, and septate ascospores [68]. However, the difference between our new species and *Paraco. magnoliae* is that the ascospores have 2 or 4 septa, without a sheath, and the penultimate cell is enlarged, while *Paraco. magnoliae* ascospores have three septa, a sheath, and a distinct guttule. Therefore, our isolate is described as a new species from *Coffea* sp. in China.

***Xenocamarosporium***  Crous and M.J. Wingf., Persoonia 34: 185 (2015)

**Notes**: *Xenocamarosporium* (*X*.) was first proposed to includethe *Camarosporium* complex by Crous et al. [78], and the type species *X. acaciae* was an asexual morph isolated from leaf spots of *Acacia mangium* in Malaysia [78]. Later, the sexual morph of *X. acaciae* was introduced by Jayasiri et al. [57] from a decaying pod of *Leucaena* sp. as a saprotrophic fungus in Thailand. The asexual characteristics of this genus are brown and globose conidiomata, brown textura angularis cells of the peridium, hyaline and smooth conidiogenous cells lining the inner conidiomatal cavity, and ellipsoidal to subcylindrical, hyaline to golden-brown, verruculose, septate conidia [78]. The sexual characteristics of this genus are brown and immersed ascomata, textura angularis cells of the peridium, filiform and septate hamathecium, bitunicate, cylindrical to cylindric-clavate asci, and hyaline to brown, cylindrical, septate ascospores, which are often enlarged at the fourth cell [57]. To date, this genus consists of only one species [22,79]. Here, we introduce one new host and country record in *Xenocamarosporium* for coffee in China.

**Figure 8:**
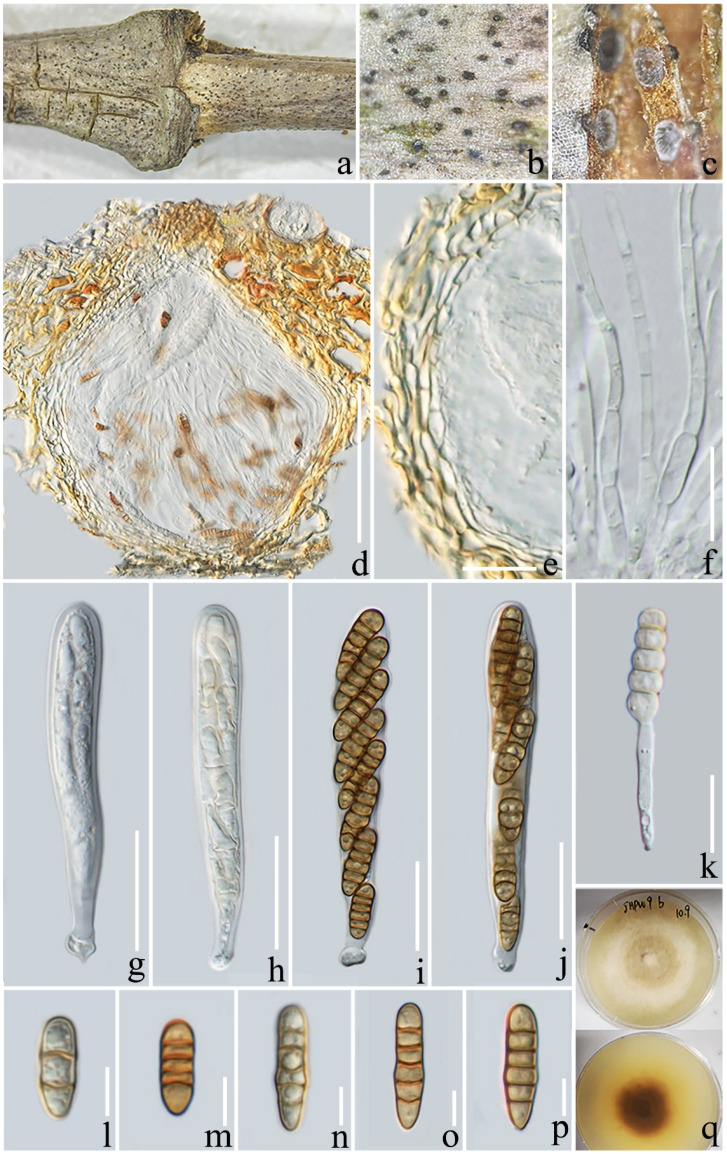
*Xenocamarosporium acaciae* (ZHKU 22-0117). (**a**,**b**) Appearance of ascomata on a decaying branch of *Coffea* sp.; (**c**,**d**) vertical section of an ascoma; (**e**) peridium; (**f**) pseudoparaphyses; (**g**–**j**) asci; (**k**) germinated ascospore; (**l**–**p**) ascospores; (**q**) culture on PDA from above and reverse (60 days). Scale bars: (**d**) = 100 µm; (**e**–**f**) = 15 µm; (**g**–**k**) = 20 µm; (**l**–**p**) = 5 µm.

***Xenocamarosporium acaciae*** Crous and M.J. Wingf., Persoonia 34: 185 (2015) (Figure 8).

Index Fungorum number: IF 812423; Faces of Fungi number: FoF 05248.

*Saprotrophic* on decaying branch of *Coffea* sp. **Sexual morph**: *Ascomata*: 100–200 × 110–200 μm ($\bar{x}$ = 177 × 169 μm, *n* = 15), immersed, subglobose, scattered, solitary, uniloculate, with short ostiole penetrating through host surface and becoming brown to black spots on the substrate. *Peridium:* 14–19 μm wide ($\bar{x}$ = 16.9 μm; *n* = 20), unequal thickness, composed of 3–4 layers of hyaline to light brown, pseudoparenchymatous cells of *textura angularis*. *Hamathecium:* 1.5–3 μm wide ($\bar{x}$ = 2.1 μm, *n* = 20), sparse, filamentous, numerous, with a distinct constricted septum. *Asci:* 60–80 × 9–11 μm ($\bar{x}$ = 71.5 × 10 μm; *n* = 20), bitunicate, 8-spored, cylindrical to cylindric-clavate, with short or absent pedicellate, apically rounded with an indistinct ocular chamber. *Ascospores:* 13–19 × 4–5.5 μm ($\bar{x}$ = 15.8 × 4.7 μm; *n* = 30), uniseriate or overlapping, hyaline to brown, cylindrical, narrower and longer at the lower cell, 2–5-septate, often enlarged at the fourth cell, conical at the top. **Asexual morph**: undetermined.

**Culture characteristics**: Ascospores germinated in PDA within 12 h. Colonies reached 50 mm diameter after two months at 25 °C, surfaces were yellow-gray to white, had margins with aerial mycelium, and were circular and flatted. The reverse side was brown to light yellow.

**Material examined**: China, Yunnan Province, Xishuangbanna, on a decaying branch of *Coffea* sp., (101°2’44’’ E, 22°31’18’’ N, 856.9 m), 15th September 2021, Li Lu, JHPW 9 (ZHKU 22-0117), ZHKUCC 22-0202 = ZHKUCC 22-0203. GenBank number; ITS: OP297805, LSU: OP297775, SSU: OP297789, *tef*1-α: OP321574, (ZHKUCC 22-0202); ITS: OP297806, LSU: OP297776, SSU: OP297790, *tef*1-α: OP321575, (ZHKUCC 22-0203).

**Notes**: In phylogeny, our strains form a clade with *Xenocamarosporium acaciae*, and the BLAST results of ITS, LSU, SSU, and *tef*1-α of our strain give 100% (MK347766), 99% (MK347983), 99.9% (MK360093), and 99.9% (MK347873) similarities with *X. acaciae*, respectively. Furthermore, the comparison of morphological characteristics of asci and ascospores shows that our isolate is highly consistent with the sexual morph of *X. acaciae* morphologically [57]. Therefore, in this study, *X. acaciae* (ZHKU 22-0117) is reported as a new host record and a country record from coffee in China.

***Paraconiothyrium fungicola*** Verkley and Wicklow, in Verkley, da Silva, Wicklow and Crous, Stud. Mycol. 50(2): 331 (2004).

Index Fungorum number: IF 500084

Synonyms: *Paracamarosporium fungicola* (Verkley and Wicklow) Wijayawardene and K.D. Hyde.

**Notes**: Wijayawardene et al. [80] transferred *Paraconiothyrium fungicola* (CBS 113269) to *Paracamarosporium* (*Paraca*.) as a new combination of *Paraca. fungicola*, since it formed a clade with *Paraca. psoraleae* (CPC 21632) in the combined gene phylogeny of ITS, LSU, and SSU. However, the phylogenetic tree of Wijayawardene et al. [80] only contained two species of *Paraconiothyrium* viz. *Paraco. estuarinum* (CBS 109850) and *Paraco. cyclothyrioides* (CBS 432.75). However, our combined gene phylogenetic analyses of ITS, LSU, SSU, *rpb*2, and *tef*1-α included all *Paraconiothyrium* and *Paracamarosporium* species, showing that *Paraco. fungicola* still clusters within *Paraconiothyrium* and is closely related to *Paraco. magnoliae* (MFLUCC 10-0278). Our results are consistent with Wang et al. [76]. Based on nucleotide comparisons, *Paraco. fungicola* (CBS 113269) is different from *Paraca. psoraleae* (CPC 21632) by 38/552 bp (6.8%) of the ITS and 3/896 bp (0.3%) of the LSU, while it is different from *Paraco. magnoliae* (MFLUCC 10-0278) in 33/524 bp (6.2%) of the ITS, and 0/899 bp (0%) of the LSU. In addition, the morphology fits with the characteristics of *Paraconiothyrium* [71,73]. Therefore, we recommend that *Paraco. fungicola* should be transferred back to the genus *Paraconiothyrium*, as first reported by Verkley et al. [75].

## 4. Discussion

In this study, four new taxa and three new records isolated from coffee are introduced based on morphological and phylogenetic analyses. In addition, a known species was transferred to *Paraconiothyrium* based on our phylogenetic analyses and the study of Wang et al. [76].

*Dictyosporiaceae* contains 17 genera and 125 species [22], but none belong to coffee-associated fungi. Herein, we introduce two new species in the genus of *Pseudocoleophoma* viz. *Pse. puerensis* and *Pse. yunnanensis*, from Yunnan Province, China. This is the first report of coffee-associated fungi in *Dictyosporiaceae* [10,74].

*Didymosphaeriaceae* contains 33 genera and 255 species [22]. To date, only five species have been reported as coffee-associated fungi in *Didymosphaeriaceae* viz. *Didymosphaeria* sp., *Montagnula donacina*, *Paraconiothyrium brasiliense*, *Phaeodothis winteri*, and *Spegazzinia meliolae* [10,74]. In this study, we introduce two new species viz. *Deniquelata yunnanensis* and *Paraconiothyrium yunnanensis*, and three new records viz. *Austropleospora keteleeriae*, *Montagnula thailandica*, and *Xenocamarosporium acaciae* in *Didymosphaeriaceae*. This is the first report of fungi belonging to *Austropleospora*, *Deniquelata*, and *Xenocamarosporium* associated with coffee.

In our multigene phylogeny, *Paraconiothyrium* is polyphyletic and paraphyletic within *Didymosphaeriaceae*, which repeats the results of previous studies [74,76,77,81]. Most species of *Paraconiothyrium* that were published earlier were mainly based on morphological taxonomy, especially asexual morphology [76,82]. In subsequent studies, some species were classified into other genera. In our phylogenetic tree, we also found the same taxonomic problems in this genus as Phukhamsakda et al. [83]. *Paraconiothyrium nelloi* and *Paraco*. *fuscomaculans* clustered in the genus *Kalmusia*, while *Paraco*. *nelloi* clustered with *K*. *italica* with high statistical support (ML/BYPP = 96/1.00). *Paraconiothyrium nelloi* (MFLU 14-0813) shows few nucleotide differences compared with *K*. *italica* (MFLUCC 13-0066), by 1/502 bp (0.2%) of the ITS, 3/857 bp (0.35%) of the LSU, and 2/995 bp (0.2%) of the SSU. The asexual morph of *Paraco*. *nelloi* was isolated from a dead twig, while *K*. *italica* was observed from the culture of PDA. Conidia of both species differ in size but are similar in shape and color [84]. *Paraconiothyrium fuscomaculans* (CBS 116.16), grouped with *K*. *longispora*, showed good statistical support (ML/BYPP = 82/0.90). Based on nucleotide comparisons, *Paraco*. *fuscomaculans* (CBS116.16) is slightly different from *K*. *longispora* (CBS 582.83) by 0/532 bp (0%) of the ITS and 1/901 bp (0.1%) of the LSU. Morphologically, *Paraco*. *fuscomaculans* and *K*. *longispora* have septate conidiogenous cells and almost the same size conidia [58,85]. In 2020, Gonçalves et al. [77] also reported *Paraco*. *nelloi* (MFLU 14-0813) and *Paraco. fuscomaculans* (CBS 116.16) that fit in the genus *Kalmusia*, and mentioned that morphological analyses of *Didymosphaeriaceae* are needed to evaluate the redisposition of *Paraconiothyrium*-like species. Therefore, in future research, we strongly recommend that these two species should be re-collected and re-identified with more collections in order to demonstrate their taxonomic positions. When studying the species of *Paraconiothyrium*, both morphology and multigene phylogeny are important, and all the *Paraconiothyrium* taxa should be included in the phylogenetic analyses. In our study, we introduce a new species of *Paraconiothyrium* based on multigene phylogeny and morphology.

We excluded the type species *Austropleospora osteospermi* in our phylogenetic analyses due to the lack of multi-gene sequence data [61]. In ITS phylogenetic analysis (not shown), *A. osteospermi* was not grouped within *Austropleospora*, thus, we believe that only the ITS gene is insufficient to distinguish intra-species in the phylogeny. The sexual morph of *A. osteospermi* is similar to *A. ochracea* [61,63]; therefore, we suggest adding multi-gene sequence data for *A. osteospermi* in future studies.

In this research, we mainly focused on coffee-associated saprotrophic fungi in Yunnan Province, China, which is one of the biodiversity hotspots in the Greater Mekong Subregion [86,87]. Saprotrophic fungi are considered one of the most active plant litter decomposers that play an important role in the cycling of carbon, nitrogen, and soil nutrients [88]. Some saprotrophic fungi can also release various chemical compounds to increase plant resistance in harsh environments [89]. In addition, some saprotrophic fungi have been investigated for plant disease control [14]. Including the fungi that are reported in this study, to date, about 40 saprotrophic fungi have been recorded from coffee [3,8,10]. Even though coffee is one of the important economic crops worldwide, very few saprotrophic fungi studies on coffee have been carried out in China, and thus, it is important to study coffee saprophytic fungi and their contribution to coffee plants. Our research increases the knowledge of coffee saprophytic fungi and provides a basis for future research on the applied aspects of coffee saprotrophic fungi.

## 5. Conclusions

In conclusion, four novel taxa belonging to *Pleosporales* that are associated with coffee were discovered. *Pseudocoleophoma puerensis* and *Pse. yunnanensis* are introduced as new species in *Dictyosporiaceae*, while *Deniquelata yunnanensis* and *Paraconiothyrium yunnanensis* are introduced as new species in *Didymosphaeriaceae*. The phylogenetic relationships among species of these three genera were also updated in this study. In addition, *Austropleospora keteleeriae* as a new host record for coffee, as well as *Montagnula thailandica*, and *Xenocamarosporium acaciae* as the new host and country records for coffee in China were reported. Furthermore, based on taxonomy and phylogenetic analyses, *Paracamarosporium fungicola* was transferred back to *Paraconiothyrium*.

## Data Availability

All the phylogenetic alignments and trees obtained are available in Figshare (doi:10.6084/m9.figshare.21260589) and TreeBASE (number 29752).

## Supplementary Materials

The following supporting information can be downloaded at: https://www.mdpi.com/article/10.3390/jof8101113/s1, Table S1: Taxa names, collection numbers, and corresponding GenBank accession numbers of the taxa used in the phylogenetic analyses. Newly generated sequences in this study are indicated in black bold. The type species are noted with ^T^ after the species name, while NA indicates the unavailability of data.
