# Supplementary material for: Description of Four Novel Species in Pleosporales Associated with Coffee in Yunnan, China
Source: J Fungi (Basel). 2022 Oct 21;8(10):1113. doi: 10.3390/jof8101113 (PMC9605522; doi:10.3390/jof8101113)
Supplement: Supplementary file 1 [file jof-08-01113-s001.zip › jof-1934473-supplementary.pdf]

Journal of *Fungi*

MDPI

Article

Description of Four Novel Species in *Pleosporales* Associated with Coffee in Yunnan, China

Li Lu [1,2,3], Samantha C. Karunarathna [1], Dong-Qin Dai [1], Yin-Ru Xiong [2,3,4], Nakarin Suwannarach [5], Steven L. Stephenson [6], Abdallah M. Elgorban [7], Salim Al-Rejaie [8], Ruvishika S. Jayawardena [2] and Saowaluck Tibpromma [1,*]

1 Center for Yunnan Plateau Biological Resources Protection and Utilization, Yunnan Engineering Research College of Biological Re-source and Food Engineering, Qujing Normal University, Qujing 655011, China
2 Center of Excellence in Fungal Research, Mae Fah Luang University, Chiang Rai 57100, Thailand
3 School of Science, Mae Fah Luang University, Chiang Rai 57100, Thailand
4 Innovative Institute for Plant Health, Zhong Kai University, Guangzhou 510550, China
5 Research Center of Microbial Diversity and Sustainable Utilization, Faculty of Science, Chiang Mai University, Chiang Mai 50200, Thailand
6 Department of Biological Sciences, University of Arkansas, Fayetteville, AR 72701, USA
7 Department of Botany and Microbiology, College of Science, King Saud University, Riyadh P.O. Box 145111, Saudi Arabia
8 Department of Pharmacology & Toxicology, College of Pharmacy, King Saud University, Riyadh P.O. Box 145111, Saudi Arabia
* Correspondence: saowaluckfai@gmail.com

Table S1. Taxa names, collection numbers, and corresponding GenBank accession numbers of the taxa used in the phylogenetic analyses. Newly generated sequences in this study are indicated in bold. The type species are noted with T after the species name, while NA indicates the unavailability of data.

Taxa names	Collection number	GenBank Accession Numbers				
		ITS	LSU	*rpb2*	SSU	*tef1-α*
Alloconiothyrium aptrootii	CBS 980.95	JX496121	JX496234	NA	NA	NA
Alloconiothyrium aptrootii T	CBS 981.95	JX496122	JX496235	NA	NA	NA
Alloconiothyrium camelliae	NTUCC 17-032-1	MT112294	MT071270	NA	MT071221	MT232967
Alloconiothyrium camelliae	NTUCC 17-032-2	MT112295	MT071271	NA	MT071222	MT232965
Alloconiothyrium camelliae	NTUCC 17-032-3	MT112296	MT071272	NA	MT071223	MT232966
Austropleospora keteleeriae	**ZHKUCC 22-0208**	**OP297801**	**OP297771**	**NA**	**OP297785**	**OP321570**
Austropleospora keteleeriae	**ZHKUCC 22-0209**	**OP297802**	**OP297772**	**NA**	**OP297786**	**OP321571**
Austropleospora keteleeriae T	MFLUCC 18-1551	MK347802	MK348021	NA	MK347910	MK360045
Austropleospora ochracea T	KUMCC 20-0020	MT799859	MT799860	NA	MT808321	MT872714
Bimuria novae-zelandiae T	CBS 107.79	MH861181	AY016356	DQ470917	NA	DQ471087
Bimuria omanensis	SQUCC 15280	MT274326	MT271820	NA	NA	MT279046
Deniquelata barringtoniae	MFLUCC 11-0257	KM213997	KM214000	NA	KM214003	NA
Deniquelata barringtoniae	MFLUCC 11-0422	JX254654	JX254655	NA	JX254656	NA
Deniquelata hypolithi T	CBS:146988	MZ064429	MZ064486	MZ078201	NA	MZ078250
Deniquelata quercina	ABRIICC 10068	MH316153	MH316157	NA	MH316155	NA
Deniquelata quercina	ABRIICC 10111	MH316154	MH316158	NA	MH316156	NA
Deniquelata vittalii	NFCCI 4249	MF406218	MF182395	NA	MF622059	NA
Deniquelata yunnanensis T	**ZHKUCC 22-0198**	**OP297803**	**OP297773**	**OP321562**	**OP297787**	**OP321572**
Deniquelata yunnanensis	**ZHKUCC 22-0199**	**OP297804**	**OP297774**	**OP321563**	**OP297788**	**OP321573**
Didymocrea sadasivanii	CBS 438.65	MH870299	DQ384103	NA	DQ384066	NA
Didymosphaeria rubi-ulmifolii T	MFLUCC 14-0023	NA	KJ436586	NA	KJ436588	NA

Didymosphaeria rubi-ulmifolii	MFLUCC 14-0024	NA	KJ436585	NA	KJ436587	NA
Didymosphaeria rubi-ulmifolii T	CBS 100299	NA	JX496124	NA	AY642523	NA
Gregarithecium curvisporum	KT922	AB809644	AB807547	NA	AB797257	AB808523
Gregarithecium sp.	MFLUCC 13-0853	KX364281	KX364282	NA	KX364283	NA
Kalmusia ebuli T	CBS 123120	KF796674	JN644073	NA	JN851818	NA
Kalmusia italica T	MFLUCC 13-0066	KP325440	KP325441	NA	KP325442	NA
Kalmusia longisporum	CBS 582.83	JX496097	JX496210	NA	NA	NA
Kalmusia longisporum	CBS 824.84	JX496115	JX496228	NA	NA	NA
Kalmusia sarothamni	CBS 116474	KF796676	KF796673	NA	KF796672	NA
Karstenula rhodostoma	CBS 690.94	NA	GU301821	NA	GU296154	GU349067
Karstenula rhodostoma	CBS 691.94	LC014559	AB807531	NA	AB797241	AB808506
Laburnicola centaureae T	MFLUCC 13-0601	KX274239	KU743192	NA	KU743193	NA
Laburnicola hawksworthii T	MFLUCC 13-0602	KU743194	KU743195	NA	KU743196	NA
Laburnicola muriformis	MFLUCC 14-0921	KU743200	KU743201	NA	KU743202	NA
Laburnicola muriformis T	MFLUCC 16-0290	KU743197	KU743198	NA	KU743199	KU743213
Letendraea cordylinicola T	MFLUCC 11-0148	NR154118	NG059530	NA	NG068362	NA
Letendraea cordylinicola	MFLUCC11-0150	KM213996	KM213999	NA	KM214002	NA
Letendraea padouk	CBS 485.70	NA	AY849951	NA	GU296162	NA
Montagnula aloes	CPC 19671	JX069863	JX069847	NA	NA	NA
Montagnula aloes T	CBS 132531	NR111757	NG042676	NA	NA	NA
Montagnula appendiculata T	CBS 109027	DQ435529	AY772016	NA	NA	NA
Montagnula bellevaliae T	MFLUCC 14-0924	KT443906	KT443902	NA	KT443904	NA
Montagnula camporesii T	MFLUCC 16-1369	MN401746	NG070946	NA	NG068418	MN397908
Montagnula chiangraiensis T	MFLUCC 17-1420	NR168864	NG068707	NA	NG070155	NA
Montagnula chromolaenicola T	MFLUCC 17-1469	NR168866	NG070948	NA	NG070157	MT235773
Montagnula chromolaenae T	MFLUCC 17-1435	NR168865	NG068708	NA	NG070156	NA
Montagnula cirsii	MFLUCC 13-0680	KX274242	KX274249	NA	KX274255	KX284707
Montagnula cylindrospora T	UTHSC DI16-208	LT796834	LN907351	NA	NA	LT797074
Montagnula donacina	HFG07004	MF967419	MF183940	NA	NA	NA
Montagnula donacina	HVVV01	KJ628375	KJ628377	NA	KJ628376	NA
Montagnula graminicola T	MFLUCC 13-0352	KM658314	KM658315	NA	KM658316	NA
Montagnula krabiensis T	MFLUCC 16-0250	NR168179	NG068826	NA	NG068385	MH412776
Montagnula puerensis T	KUMCC 20-0225	MW567739	MW575866	NA	MW575864	MW573959
Montagnula puerensis	KUMCC 20-0331	MW567740	MW575867	NA	MW575865	MW573960
Montagnula saikhuensis	MFLUCC 16-0315	KU743209	KU743210	NA	KU743211	NA
Montagnula scabiosae T	MFLUCC 14-0954	KT443907	KT443903	NA	KT443905	NA
Montagnula thailandica	**ZHKUCC 22-0206**	**OP297807**	**OP297777**	**NA**	**OP297791**	**OP321576**
Montagnula thailandica	**ZHKUCC 22-0207**	**OP297808**	**OP297778**	**NA**	**OP297792**	**OP321577**
Montagnula thailandica T	MFLUCC 17-1508	MT214352	NG070949	NA	NG070158	MT235774
Neokalmusia brevispora	KT 1466	NA	AB524600	AB539099	AB524459	NA
Neokalmusia brevispora	KT 2313	NA	AB524601	AB539100	AB524460	NA
Neptunomyces aureus	CMG12	MK912121	NA	NA	NA	MK948000
Neptunomyces aureus	CMG13	MK912122	NA	NA	NA	MK948001
Neptunomyces aureus	CMG14	MK912123	NA	NA	NA	MK948002
Paracamarosporium fagi	CPC 24892	KR611887	KR611905	NA	NA	NA
Paracamarosporium fagi	CPC 24890	KR611886	KR611904	NA	NA	NA
Paracamarosporium hawaiiense	CPC 12268	DQ885896	NA	NA	EU295656	NA
Paracamarosporium hawaiiense T	CBS 120025	JX496027	JX496140	NA	EU295655	NA

Paracamarosporium leucadendri T	CBS:123027	EU552106	NA	NA	NA	NA
Paracamarosporium mamanes T	CBS 120031	DQ885900	NA	NA	NA	NA
Paracamarosporium psoraleae T	CPC 21632	KF777143	KF777199	NA	NA	NA
Paracamarosporium tamaricis	MFLUCC 15-0494	KU900326	KU900298	NA	NA	NA
Paracamarosporium tamaricis	MFLUCC 15-0495	KU900327	KU900299	NA	NA	NA
Paraconiothyrium ajrekarii	NFCCI 4810	MT372906	MT372905	NA	NA	NA
Paraconiothyrium archidendri	MFLUCC 17-2429	MK347757	MK347974	NA	MK347863	MK360044
Paraconiothyrium archidendri	CBS 168.77	JX496049	JX496162	NA	NA	NA
Paraconiothyrium babiogorense T	CBS 128292	MH864845	MH876291	NA	NA	NA
Paraconiothyrium camelliae	NTUCC 18-096	MT112293	MT071269	NA	MT071220	MT277330
Paraconiothyrium cyclothyrioides	CBS 972.95	JX496119	JX496232	NA	AY642524	NA
Paraconiothyrium estuarinum	CBS 109850	JX496016	JX496129	NA	AY642522	NA
Paraconiothyrium fuscomaculans	CBS 116.16	MH854649	MH866170	NA	EU754098	NA
Paraconiothyrium fungicola T	CBS 113269	JX496020	JX496133	NA	AY642527	NA
Paraconiothyrium hakeae	CBS 142521	KY979754	KY979809	KY979847	NA	NA
Paraconiothyrium iridis	CBS:146036	MT223827	MT223919	MT223695	NA	NA
Paraconiothyrium lini	CBS 253.92	NA	GU238093	NA	GU238221	NA
Paraconiothyrium lycopodinum T	CBS 134705	NA	NG_058029	NA	NA	NA
Paraconiothyrium maculicutis	CBS 101461	NA	EU754200	NA	EU754101	NA
Paraconiothyrium magnoliae	MFLUCC 10-0278	KJ939280	KJ939283	NA	NA	NA
Paraconiothyrium nelloi T	MFLU 14-0813	KP711360	KP711365	NA	KP711370	NA
Paraconiothyrium polonense T	CBS:134153	NA	KF700360	NA	NA	NA
Paraconiothyrium rosae T	MFLU 15-1115	MG828932	MG829041	NA	MG829147	NA
Paraconiothyrium salinum T	CMG 49	MN369540	NA	NA	NA	MN380481
Paraconiothyrium salinum	CMG 50	MN369541	NA	NA	NA	MN380482
Paraconiothyrium thysanolaenae T	MFLUCC 10-0550	KP744453	KP744496	NA	KP753959	NA
Paraconiothyrium tiliae	CBS 265.94	NA	EU754139	NA	EU754040	NA
Paraconiothyrium yunnanensis T	**ZHKUCC 22-0196**	**OP297797**	**OP297767**	**OP321560**	**OP297781**	**OP321566**
Paraconiothyrium yunnanensis	**ZHKUCC 22-0197**	**OP297798**	**OP297768**	**OP321561**	**OP297782**	**OP321567**
Paramassariosphaeria anthostomoides	MFLU 16-0172	KU743206	KU743207	NA	KU743208	NA
Paramassariosphaeria anthostomoides	CBS 615.86	MH862005	MH873693	NA	GU205246	NA
Paraphaeosphaeria angularis T	CBS 167.70	JX496047	MH871317	NA	NA	NA
Paraphaeosphaeria arecacearum	CBS 158.75	JX496043	JX496156	NA	NA	NA
Paraphaeosphaeria camelliae	NTUCC 18-095-1	MT112291	MT071267	MT743278	MT071218	MT743269
Paraphaeosphaeria camelliae	NTUCC 18-095-2	MT112292	MT071268	MT743279	MT071219	MT743270
Paraphaeosphaeria michotii	MFLUCC-13-0349	KJ939279	KJ939282	NA	KJ939285	NA
Paraphaeosphaeria michotii	CBS 652.86	JX496103	JX496216	GU456351	GQ387520	NA
Paraphaeosphaeria minitans	CBS 111750	JX496017	JX496130	NA	NA	NA
Paraphaeosphaeria minitans	CBS 861.71	MH860382	NA	NA	AY642526	NA
Paraphaeosphaeria neglecta	CBS 119637	JX496025	JX496138	NA	NA	NA
Paraphaeosphaeria neglecta	CBS 124078	MH863348	MH874872	NA	NA	NA
Paraphaeosphaeria parmeliae	CBS 131728	KP170654	KP170722	NA	NA	NA
Paraphaeosphaeria pilleata	CBS 102207	JX496013	JX496126	NA	NA	NA
Paraphaeosphaeria rosae	MFLUCC 17-2547	MG828935	MG829044	NA	MG829150	NA
Paraphaeosphaeria rosicola	MFLU 18-0108	MG828938	MG829047	NA	MG829153	NA
Paraphaeosphaeria sardoa	CBS 501.71	MH860235	MH872003	NA	NA	NA
Paraphaeosphaeria sp.	MFLUCC 15-0450	KX965729	KX954398	NA	KX986342	NA
Paraphaeosphaeria sp.	CBS 101464	JX496012	JX496125	NA	NA	NA

Paraphaeosphaeria spartii	MFLU 14 C0810	KP711357	KP711362	NA	KP711367	NA
Paraphaeosphaeria sporulosa	CBS 132.26	MH854865	MH866362	NA	NA	NA
Paraphaeosphaeria sporulosa	CBS 391.86	JX496082	JX496195	NA	NA	NA
Paraphaeosphaeria sporulosa	CBS 105.76	JX496014	JX496127	NA	NA	NA
Paraphaeosphaeria verruculosa	CBS 263.85	MH861879	MH873567	NA	NA	NA
Paraphaeosphaeria viciae	MFLU 15-1231	NR_158840	KY397947	NA	KY397948	NA
Paraphaeosphaeria viridescens T	CBS 854.73	JX496085	JX681076	NA	NA	NA
Paraphaeosphaeria xanthorrhoeae T	CBS 142164	KY979738	KY979793	KY979845	NA	NA
Periconia pseudodigitata T	KT1395	LC014591	AB807564	NA	AB797274	AB808540
Periconia pseudodigitata	KT1195A	LC014590	AB807563	NA	AB797273	AB808539
Pseudocamarosporium africanum T	STE-U 6316	EU295650	NA	NA	EU295654	NA
Pseudocamarosporium corni T	MFLUCC 13-0541	KJ747048	KJ813279	NA	KJ819946	NA
Pseudocamarosporium cotinae T	MFLUCC 14-0624	KP744460	KP744505	NA	KP753964	NA
Pseudocamarosporium lonicerae T	MFLUCC 13-0532	KJ747047	KJ813278	NA	KJ819947	NA
Pseudocamarosporium propinquum	MFLUCC 13-0544	KJ747049	KJ813280	NA	KJ819949	NA
Pseudocoleophoma bauhiniae T	MFLUCC 17-2280	MK347735	MK347952	NA	MK347843	MK360075
Pseudocoleophoma calamagrostidis T	KT3284	LC014592	LC014609	NA	LC014604	LC014614
Pseudocoleophoma flavescens	CBS 178.93	NA	GU238075	NA	GU238216	NA
Pseudocoleophoma polygonicola T	KT731	AB809634	AB807546	NA	AB797256	AB808522
***Pseudocoleophoma puerensis* T**	**ZHKUCC 22-0204**	**OP297799**	**OP297769**	**NA**	**OP297783**	**OP321568**
Pseudocoleophoma puerensis	**ZHKUCC 22-0205**	**OP297800**	**OP297770**	**NA**	**OP297784**	**OP321569**
Pseudocoleophoma rusci T	MFLUCC 16-1444	NR_170045	NG_073840	NA	NG_070346	NA
Pseudocoleophoma typhicola T	MFLUCC 16-0123	KX576655	KX576656	NA	NA	NA
***Pseudocoleophoma yunnanensis* T**	**ZHKUCC 22-0200**	**OP297795**	**OP297765**	**NA**	**OP297779**	**OP321564**
Pseudocoleophoma yunnanensis	**ZHKUCC 22-0201**	**OP297796**	**OP297766**	**NA**	**OP297780**	**OP321565**
Pseudocoleophoma zingiberacearum T	NCYUCC 19-0052	MN615939	MN616753	NA	NA	MN629281
Pseudoconiothyrium broussonetiae	CBS 145036	MK442618	MK442554	NA	NA	MK442709
Pseudodictyosporium elegans T	CBS 688.93	DQ018099	DQ018106	NA	DQ018084	NA
Pseudodictyosporium indicum	CBS 471.95	DQ018097	NA	NA	NA	NA
Pseudodictyosporium wauense	NBRC 30078	DQ018098	DQ018105	NA	DQ018083	NA
Pseudodictyosporium thailandica T	MFLUCC 16-0029	NR_154347	NG_059688	NA	NG_063611	KX259526
Spegazzinia deightonii	MFLUCC 20-0002	MN956768	MN956772	NA	MN956770	MN927133
Spegazzinia deightonii	yone 212	NA	AB807582	NA	AB797292	AB808558
Stagonospora paludosa	CBS 135088	NR155787	KF251760	NA	NA	NA
Stagonospora pseudocaricis	CBS 135132	KF251259	KF251762	NA	NA	NA
Xenocamarosporium acaciae	**ZHKUCC 22-0202**	**OP297805**	**OP297775**	**NA**	**OP297789**	**OP321574**
Xenocamarosporium acaciae	**ZHKUCC 22-0203**	**OP297806**	**OP297776**	**NA**	**OP297790**	**OP321575**
Xenocamarosporium acaciae T	CPC 24755	KR476724	KR476759	NA	NA	NA
Xenocamarosporium acaciae	C354	MK347766	MK347983	NA	MK347873	MK360093
